# Life cycle inventories of waste management processes

**DOI:** 10.1016/j.dib.2018.05.067

**Published:** 2018-05-19

**Authors:** Melanie Haupt, Thomas Kägi, Stefanie Hellweg

**Affiliations:** a ETH Zurich, Institute of Environmental Engineering, John-von-Neumann-Weg 9, CH- 8093 Zurich, Switzerland; b Carbotech AG, Gasometerstrasse 9, CH-8005 Zurich, Switzerland

## Abstract

To allow for an up-to-date and geographically specific life cycle assessment, updated and regionally specific life cycle inventories are crucial. This data article present up-to-date life cycle inventories of several collection, sorting and recycling processes of municipal solid waste fractions for life cycle assessments of waste management systems. In total, 190 life cycle inventories for processes within municipal solid waste management were either newly developed or adapted from existing datasets. The data for 51 recycling processes has been collected to update existing processes or create new process models. Two modules for biogenic processes were taken from literature and 10 processes were modeled based on the existing ecoinvent processes with minor adjustments [1]. The substitution of 36 materials from recycling processes was modeled. In addition, the thermal treatment of 12 waste fractions was modeled within 84 life cycle inventories compromising the thermal waste treatment and the recovery and recycling of recovered fractions from fly and bottom ash. The assumptions and the modelling of the waste treatment processes are described. All life cycle inventory datasets which were newly created, updated or modified compared to the original dataset are described and provided as Excel table. The data are associated with the research article "Modular Life Cycle Assessment of Municipal Solid Waste Management” [2].

**Specifications Table**

**Table:** 

Subject area	*Waste Management*
More specific subject area	*Life Cycle Assessment*
Type of data	*Excel table*
How data was acquired	*Collected industrial data*
Data format	*Life Cycle Inventories which are modelled from industrial data*
Experimental factors	–
Experimental features	–
Data source location	*Switzerland (Data from Swiss Recycling Association, sometimes literature values from Europe were used in modelling)*
Data accessibility	*The Life Cycle Inventories are attached as Supplementary Material to this paper (in an excel sheet).*
Related research article	*Haupt et al.*[2].

**Value of the data**

● Life Cycle Inventories for 190 waste treatment processes in industrial countries enable in-depth case studies on waste management systems
● Up-to-date life cycle inventories for recycling processes enable a comparison of open- and closed-loop recycling pathways
● The split of the inventories into collection, sorting and recycling steps allow for adaption to individual waste management systems and systems optimization.

## 1 Data

The Swiss municipal solid waste management system comprises several hundred processes such as separate and mixed collection, sorting of recyclables, pre-treatments, recycling, thermal treatments, bottom and fly ash treatments, and substitution processes for materials and energy. For a case study on the Swiss municipal solid waste management system in [2], life cycle assessments of more than 500 processes were needed. For this, life cycle inventories for 190 process were newly modelled or adapted from previous data sources. The new life cycle inventories, which are presented in this data article, enabled the environmental assessment on a waste-fraction specific level for the current situation and in various scenarios. The data can also be used and combined flexibly to model other waste management systems.

## 2 Experimental design, materials, and methods

**Fig. 1:**
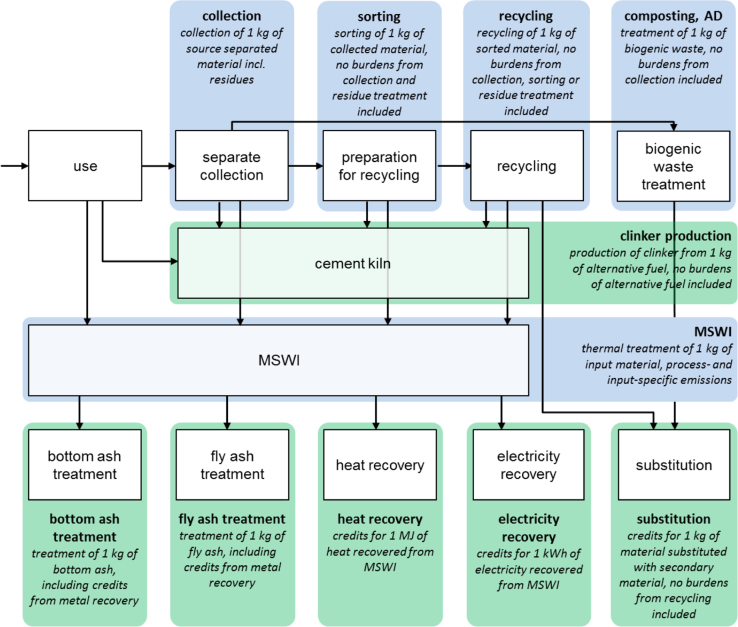
Overview of all life cycle inventories, their system boundaries and functional units. AD = anaerobic digestion, MSWI = municipal solid waste incineration. Green boxes mark processes where normally credits result as they include the benefits from substitution, blue processes contain the burdens of material treatments.

The modular setup of the life cycle assessment requires input-oriented functional units, i.e. the function unit is related to the input of the process, of all processes [2]. Fig. 1 describes the system boundaries and functional units of all module types (e.g. collection, sorting, recycling). The life cycle inventories do not include the production, sorting, or upgrading steps of the entering waste material streams, i.e. if the inventories are used for other projects, the waste material inputs and related burdens have to be added manually. As a background database for the inventories, ecoinvent v3.3 (cut-off) is used [1].

The uncertainty of most data collected from industry is unknown, as in general only one value was available without any knowledge on the distribution. Therefore, the uncertainties were estimated based on the methodology described in Weidema et al. [3]. In the inventories provided, the uncertainty is given as basic uncertainty factors (variances of the underlying normal distribution to the lognormal distribution, table provided in Weidema et al. [3]) and the pedigree matrix. The data reliability and completeness and the temporal, geographical and technical correlation were estimated by the authors based on expert interviews, literature reviews and observations on the Swiss waste management system.

All inventories are provided in the data containing appendix. Some inventories for recycling processes are also provided in the original project report [4], but were adapted to the modular structure for this project. Therefore, all inventories are described below.

## 3 Life cycle inventories

### 3.1 Life cycle inventories for cardboard recycling

The cardboard recycling to corrugated board was previously not modelled for Switzerland but available only as European average in 2006. For fiber based processes, geographical representation is of high importance as each manufacturing process tends to be unique in the set of processes and the raw material mix [5]. Corrugated board consists of linerboard (the outer media, also called testliner if made from recycled material and kraftliner, if primary materials are used) and fluting medium. The inventories for both testliner and fluting medium have been newly set-up for cardboard recycling in Switzerland. A small share of waste cardboard (0.1%, based on Haupt et al. [6]) was used in 2012 to produce insulation material for buildings. The related production process has been newly modelled based on industrial data and is provided as individual life cycle inventory.

#### 3.1.1 Collection of waste cardboard

The local waste cardboard collection varies between the communities. It can either be collected separately or mixed with waste paper. While in some communities the waste cardboard is collected at the curbside, other municipalities rely on central collection points. After the local collection, the waste cardboard is transported by lorry or train either directly to sorting or to intermediate recycling plants. The local collection was modeled assuming a transport of 25 km with a waste collection vehicle. In addition, 100 km of transport by lorry and 50 km by freight train have been added [7].

#### 3.1.2 Sorting of waste cardboard

Before the collected waste cardboard enters the recycling process, it is sorted from contaminants of the collection and classified by waste paper type. The existing ecoinvent v.3.3 (cut-off) process “treatment of waste paper, unsorted, sorting {CH}” was used as a proxy for the sorting process of cardboard assuming that the energy need, auxiliary materials and waste content were similar. However, all the waste material flows were replaced with the values reported in the material flow analysis in Haupt et al. [6].

#### 3.1.3 Recycling of waste cardboard and production of secondary cardboard

The process of waste cardboard recycling involves mixing used cardboard with water and chemicals to break it down, before it is chopped and heated, to split it into strands of cellulose. The resulting pulp is strained through screens, which remove glue or plastic that may still be in the mixture, and mixed with water. Pulp from recovered fibers used to produce corrugated packaging does not undergo a de-inking process. The so-called rejects (non-fibrous materials) are separated in the course of dissolving the waste paper in the pulper. Additionally, a sophisticated sieving method cleans the waste paper, until the fibrous material is finally relieved of residues. The rejects are collected, dried, sorted and fed to the thermal utilization system.

Testliner, i.e. linerboard from recycled materials, mostly consists of two plies of paper. Depending on the type of testliner, the fiber composition can be different in each layer. In general, a better grade of mix is used for the upper layer for reasons of appearance and strength. In order to increase its strength, testliner receives a surface treatment in the size press. This involves the application of a starch solution to one or both sides of the sheet. Fluting medium can be a one-ply or two-ply product. Usually, a size press treatment with a starch solution is applied in-line on the paper machine in order to obtain sufficient strength and stiffness properties. The most common surface treatment of recovered fiber based corrugated board material is done by a size press. Essentially a size press comprises two revolving rubber covered rolls, pressed together, through which the paper web passes. In the nip formed by the rolls there is a starch solution. The paper absorbs some of this solution, is pressed between two rolls and goes into the “after dryer“ section of the paper machine in order to evaporate excess water absorbed from the starch solution in the size press. After the paper machine, there is a slitter winder where the big jumbo reel from the paper machine is rewound and cut down to customer reel formats according to customer orders. Finally the reels are weighed, marked, labelled and prepared for shipment to the customer, the corrugated board industry.

Data for the recycling process were derived from Model Group, Switzerland [7]. Lacking data on chemicals and auxiliary materials were completed with data from the ecoinvent dataset “linerboard {RER} treatment of recovered paper to testliner”. Data from Model Group could not be distinguished between testliner and fluting medium with regard to energy and water use. It is assumed that the energy and water use is very similar. This assumption is further supported by the existing inventories in ecoinvent (“linerboard {RER} treatment of recovered paper to testliner” and “fluting medium {RER} treatment of recovered paper to wellenstoff”).

#### 3.1.4 Substitution

Secondary fibers as output of the cardboard recycling processes are assumed to substitute primary cardboard fibers. The substitution of insulation was modeled material specific: insulation from cardboard replaces insulation material made from primary cellulose to ensure substitutability of the insulation materials in their applications. Losses in the production process (e.g. fibers too short for recycling) are assessed in the material flow analysis, i.e. are directed towards their final treatment which is modelled separately.

### 3.2 Life cycle inventories for paper recycling

Paper can be recycled to newsprint or graphical paper. The ecoinvent dataset for newsprint production in Switzerland dates back to 2000. The inventories have therefore been updated for the recycling process in Switzerland. A small share of waste paper (1%, based on Haupt et al. [6]) was, in 2012, used to produce insulation material for buildings. The related production process has been newly modelled based on industrial data and is provided as individual life cycle inventory.

#### 3.2.1 Collection of waste paper

For the collection of waste paper from households, the same transport distance as for the cardboard recycling was assumed (150 km based on “waste cardboard, collected {CH}”, see chapter 1.1.1). The share of transport by railway is given in the annual reports of both Swiss companies, resulting (weighted according to production volume) in 116 km transport by lorry and 34 km transport by rail [8], [9].

#### 3.2.2 Recycling of waste paper to newsprint

In Switzerland, two large newsprint paper mills were in operation. Both of them are integrated companies, producing their own deinked pulp from waste paper and also their own mechanical pulp from primary materials. Main raw material in both cases is waste paper (post-consumer or production residues). Waste paper is collected from small businesses and households and brought either directly to the paper recycling company or to intermediate recyclers and sorters.

Besides recovered paper, softwood, either as thinning wood from the forest, or as chips from wood industry, is the main raw material used for the production of newsprint in Switzerland. Besides wood, recovered paper and water, several chemicals are used within the whole production process. For chemicals which were not part of the ecoinvent database, the modules "chemicals organic, at plant" and „chemicals inorganic, at plant“ are used as a first approximation. The transport distances for the different raw and auxiliary materials used within the process was based on Hischier [10]. The share of rail and road was known for both companies based on their annual reports [8], [9] and implemented accordingly.

Collected waste paper is not only processed to newsprint but also to graphical paper. The largest difference between the production of newsprint and graphical paper is the addition of fillers and primary pulp. The mix of chemicals is almost identical for both processes (based on internal reports). The recycling process to newsprint is, however, representative for 91% of the waste paper recycled in Switzerland and was assumed as sole pathway here.

The process of waste paper recycling starts with the loading of waste paper onto transport belts on which they enter the pulper. In the pulper, water and chemicals are added to break the paper down to pulp. The large non-fibrous contaminants are then removed (e.g. staples, plastic, glass). The fibers are progressively cleaned using a deinking procedure and the resulting pulp is filtered and screened again to make it suitable for papermaking. After the ink is removed, the fibers may be bleached and treated by a secondary deinking step before it enters the paper producing process. The production of newsprint from waste paper further includes the paper machine, on-site energy production as well as flue gas cleaning technology and waste water treatment plant. A detailed process description can be found in Hischier [10]. The life cycle inventory presented includes the thermal treatment of residues from the recycling process in the internal furnace.

##### 3.2.2.1 Data sources

Data for the recycling process was derived from the annual reports of the two paper producing companies in Switzerland (Perlen Papier AG and Utzenstorf Papier AG) and inventories were then finalized in close collaboration with the industrial partners. The processes used in these two companies are similar, but the mix of chemicals used differs significantly. The presented inventory represents a production volume weighted average of the two companies and describes the recycling of waste paper into newspaper. Some chemicals are not represented in the database and could therefore not be included specifically. Instead, the chemicals were grouped into organic and inorganic chemicals and included in the analysis as sum parameter.

The energy production located on paper mill sites is not only based on internal residues but also includes residues from other processes and therefore acts as waste treatment. There is, however, no matching biomass flow modelled in the ecoinvent database. Therefore, the inputs are included as dummy processes. This allows linking them with the respective biomass flows of the ecoinvent database as soon as they are available. Emissions from the utilization of these inputs in energy production are, however, included in the inventory.

No data was available on the infrastructure of the paper mill. Therefore, the data and inventory of Hischier [10] was used and the uncertainty adjusted accordingly.

##### 3.2.2.2 Emissions

Values for the most important air (fossil CO2, NOx, SO2, TSP) and water (BOD, COD, tot-N, tot-P, etc.) emissions are taken from the questionnaires filled in by both industries. Further emissions to air are estimated based on the fuels used within the internal furnaces of the different paper mills and the inventories of the respective heating systems (in conformity with Hischier [10] and Dones et al. [11]). The values from the different types of waste are based on the indications in the examined questionnaires and annual reports [8], [9].

##### 3.2.2.3 Wastes

All data on wastes was sourced from the companies. The treatment thereof was estimated in discussion with the industry and integrated in the inventory.

#### 3.2.3 Substitution

Secondary fibers as output of the paper recycling processes are assumed to substitute primary fibers. The substitution of insulation was modeled material specific: insulation from paper replaces insulation material made from primary cellulose to ensure substitutability of the insulation materials in their applications. Losses in the production process (e.g. fibers too short for recycling) are assessed in the material flow analysis, i.e. are directed towards their final treatment which is modelled separately.

### 3.3 Life cycle inventories for glass recycling

Container glass for food and beverages is made of soda-lime glass and constitutes 50–60% of the total amount of glass produced [48], but a much larger percentage of the non-durable glass that becomes waste after a single use. Therefore, only non-durable glass is considered here. Glass cullet from soda-lime glass is recycled to green packaging glass, and is used in the foam glass plate, for gravel production, or to produce glass sand.

#### 3.3.1 Collection of packaging glass

The local collection of packaging glass is organized by the communities. Glass cullet is either collected as color separated fractions or mixed-color cullet [6], [12]. From the local collection, the glass cullet is transported by lorry or train either to a sorting facility or directly to a packaging glass production plant. The life cycle inventory of collection is based on Stettler et al. [12].

#### 3.3.2 Reuse of glass packaging

A small fraction of the glass consumed at households is reused after an industrial washing process. The washing process was modelled according to Kägi and Dinkel [13] and scaled to 1 kg of glass from households.

#### 3.3.3 Sorting of glass cullet

The collected glass cullet needs to be sorted for its use in the glass or foam glass production. The sorting of cullet eliminates impurities (plastics, metals, ceramics, cullet of other colors in the case of glass packaging production). As no primary data was available, this module was based on the ecoinvent process “treatment of waste glass from unsorted public collection, sorting, RER” (v3.3, cut-off). The dataset is originally based strongly on BUWAL [14], i.e. Swiss inventories, and is complemented with data from Germany [15]. The following changes have been made: First, the dataset was scaled to 1 kg of input (instead of 1 kg of output). Second, all the waste treatments were taken out of the process’ inventory as they have been considered separately. Their treatment can then be modelled case-specific based on a material flow analysis. Only for the CH dataset, in addition, the electricity and water consumption was adapted to Switzerland. The color-sorting process for glass cullet as in place in Germany was approximated with the same sorting process as described above.

#### 3.3.4 Production of green packaging glass

The glass cullet directly replaces raw materials in the packaging glass or foam glass production. The use of glass cullet reduces the release of CO_2_ from carbonates and leads to a lower energy consumption in the melting process (about 2% to 3% per 10% of glass cullet). Limiting factors for the use of glass cullet is the desired color quality. After the sorting and crushing, the glass cullet is melted together with the new raw materials at temperatures of up to 1580 °C. The glowing, viscous, melted glass is first preformed and then blown with compressed air to ready-to-use glass containers, before the bottles move into the so-called cooling furnace. Here, they are carefully cooled and then coated with a special protective spray to protect them from scratches and to increase the breaking strength. At last, the glass packaging is tested several times for cracks, inclusions, deformations or other defects optically, mechanically and electronically.

Data on the raw material and the energy used in the production of green glass is available for Switzerland but cannot be used for confidentiality reasons. The data has, however, been used to partly update and adapt the existing ecoinvent inventory “packaging glass, green {DE}”, assuming similar production standards for Switzerland as for Germany. Scenarios for 100% primary resource and 100% glass cullet were derived from the data set of the average CH glass production considering the impacts of glass cullet on the energy demand. The data set for the average CH glass production corresponds to 84% glass cullet and 16% primary resources.

#### 3.3.5 Production of secondary foam glass plate or gravel

Besides the recycling to packaging glass, glass cullet can also be used for the production of foam glass plates and gravel. Glass cullet are used for the Swiss production of foam glass gravel or exported for the production of insulation materials in neighboring countries. The collection and sorting of glass cullet for the production of foam glass was assumed to be similar as for the glass production, i.e. the modules described above are used. For foam glass gravel, the sorted glass cullet are crushed and run through a multi-stage separation and comminution process. Subsequently the up to 10 mm large glass pieces are milled to fine glass flour to which minerals activators are added. Afterwards, continuous ovens are used for sintering and foaming the glass flour at temperatures around 900 °C. A hot foam glass plate leaves the oven at 300 to 400 °C. The very rapid cooling causes stress cracking, which causes the plate to disintegrate into 3 cm to 7 cm gravel grains.

For foam glass plates, the process is similar with the exception of a controlled and slow cooling process in a drawing furnace and differences in the composition of raw materials. The controlled cooling process, however, leads to a substantially higher energy demand for the production of foam glass plates compared to gravel. After cooling, a permanent negative pressure of approx. 0.5 bar is produced in the cell interior, as a result of which the thermal conductivity is additionally reduced. Data for the foam glass gravel process were derived from an existing EPD of foam glass gravel [16], considering Swiss conditions for the standard of waste treatment, transports and electricity supply.

Data for the foam glass plate process were derived from the ecoinvent inventory, but updated and complemented with data from an existing EPD of foam glass plates [17]. The electricity mix was adjusted for the foam glass plates to represent the generic RER situation (no production of foam glass plates in Switzerland).

#### 3.3.6 Production of sand from glass cullet

Glass cullet can also be crushed to glass sand. Data for glass sand crushing was approximated with the existing ecoinvent v3.3 process “limestone, crushed, for mill {CH}”.

#### 3.3.7 Substitution

Foam glass, as gravel or plates, is used as insulation material in the building sector where it is assumed to replace 25% primary foam glass plates and 75% XPS insulation [12]. Generally, the substitution factors for insulation material take the thermal conductivity and the density into account resulting, for example, in a factor of 0.21 for the substitution of XPS by foam glass (1 kg of foam glass plate replaces 0.21 kg of XPS.

### 3.4 Life cycle inventories for plastic recycling

While polyethylene-terephthalate (PET) bottles from beverages have been collected since 1990 in Switzerland, the recycling of plastic is relatively new. In parallel to the PET collection, PE from milk bottles has been recycled for some years without yielding high collection rates. In 2012, only minor amounts of PET and PE were therefore collected [6]. In the last years, however, several separate collection systems have been built up collecting either plastic bottles of mixed polymers or mixed packaging plastics in general. Some even include the collection of beverage cartons, which are not collected individually and are utilized in cardboard recycling companies.

The following chapters are therefore split into PET bottles recycling, mixed plastic recycling and the recycling chain of beverage cartons.

#### 3.4.1 Life cycle inventories for PET bottle recycling

The recycling of PET is one of the oldest recycling schemes and in place since 1990 [18] and collection rates are high (85%) in international comparison. The PET recycling system has regularly been assessed using life cycle assessment [19], [20] and was updated for the presented inventories. The collection system for PET bottles is well established with collection points in public spaces, at retailers and at municipal collection centers. Collected material is sorted in one of the five sorting centers in Switzerland or exported for recycling. After sorting out the residues and color-sorting, the material enters either the open-loop recycling to PET fibers (multi-colored PET) or the closed-loop recycling (blue and transparent PET).

##### 3.4.1.1 Collection of waste PET bottles

PET bottles are collected in four channels: from communities, at retailers, at distributers, and at facultative sites such as schools, train stations and parks. Data were derived from PET Recycling Schweiz (PRS) that is responsible for the comprehensive logistic-net [21]. Overall, there are more than 40 subcontractors involved in the PET bottle collection.

##### 3.4.1.2 Sorting of waste PET bottles

The collected PET bottles are sorted by colors in five sorting centers in Switzerland and compressed to bales of 200 kg to 300 kg for transport and further processing. Foreign substances are sorted out and disposed of in cement kilns or municipal solid waste incineration plants. Data were derived from involved sorting facilities. The data is newly added to the ecoinvent database (version 3.4).

##### 3.4.1.3 Production of secondary PET granulate

After sorting, the PET bales (blue/transparent and green/multicolored) are processed according to the United Resource Recovery Corporation (URRC)-procedure in one of two recycling plants in Switzerland. First, the wire around the PET bales is cut away and the bottles are separated again. The PET bottles pass through a metal separator which also separates aluminum-containing labels. The bottles are then shredded with knives to 12 mm size in the mill. The so-called flakes are fed from above into the air separator separating label residues from the PET flakes. The label residues end up in the municipal solid waste incineration. Purified from the labels, the flakes enter a pool of water where the bottle caps, made of PE, are separated in a sink-float process. The caps float on top whereas the PET flakes, which are heavier than water, sink to the bottom allowing an easy separation from the PET flakes and later PE recycling. The PET flakes are treated with 50% sodium hydroxide and the flakes-liquor mixture is heated to approximately 200 °C. The liquor dissolves the PET surface layer and removes even the SiOx coating of the bottle-inside. The purified PET flakes are washed with water and then dried. The PET flakes have now such a high quality that they may come into contact with food again. There are two recycled PET grades produced in Switzerland: amorphous PET and bottle-grade PET. For bottle-grade PET, the viscosity of the recycled flakes is increased to a comparable level of primary PET by solid state polymerization (SSP). Data were derived from involved recycling facilities [22].

##### 3.4.1.4 Substitution

While bottle-grade PET is assumed to substitute PET in the bottle production, amorphous PET is used to produce trays, other packaging material and PET fibers. Amorphous and bottle-grade PET are assumed to substitute primary PET 1:0.95 and 1:1, respectively, based on literature and expert interviews [2].

#### 3.4.2 Life cycle inventories for mixed plastic recycling

##### 3.4.2.1 Collection of plastic waste

Consumers can deposit empty plastic bottles at retailers selling plastic bottles or at waste collection sites. In some municipalities the plastic waste is picked up directly at the curbside [23]. Data for the collection of plastic waste in Switzerland were based on some of the PET collection data [21], as these include the same retro-logistic streams. Data for the collection of plastic waste in Europe were extrapolated from PE bottles collection in the USA [24].

##### 3.4.2.2 Sorting of plastic waste

The collected plastic waste is sorted in sorting centers in Switzerland and compressed to bales of 200 kg to 300 kg for transport and further processing. Data were derived from involved sorting facilities. Impurities from collection are sorted out and are assumed to be treated in municipal solid waste incineration or utilized in cement kilns [23].

##### 3.4.2.3 Production of secondary plastic granulate

The waste plastic is further sorted and cleaned in a first step in order to remove any unwanted debris. The plastic then needs to be further sorted, so that the pure fractions are present. Nowadays, HDPE is the main plastic that is further processed to secondary granulate, but also low-density polyethylene (LDPE), polypropylene (PP), polystyrene (PS) and PET are further processed depending on the recycling facility [25]. The presence of other plastic polymers is detrimental for the quality of the secondary production. HDPE has a lower specific density than PET, meaning that these plastic polymers can be separated by sink-float separation. However, HDPE has a similar specific density to PP. In this case, Near Infrared Radiation (NIR) techniques can be used, unless the plastic is too dark and absorbs the infrared waves. Following the sorting, the waste plastic is shredded and melted to further refine the polymer. The polymer is then cooled into pellets which can be used in manufacturing. Data were derived from Tonner [26].

##### 3.4.2.4 Substitution

The material substitution benefits from recycled plastics were modeled using three quality classes. High and medium quality granulates were assumed to substitute 90% and 70%, respectively, of the corresponding primary material (polypropylene, high and low density polyethylene and high impact polystyrene) [23]. The low quality granulates were assumed to substitute wood (50% of granulates, substitution by weight) or concrete (50% of granulates, by volume) in applications like pallets, park benches and grass pavers [23], which results in crediting approximately 7% to 14% of the respective primary plastic materials (in terms of climate change).

For PET sorted out from the mixed plastic recycling, high and medium granulate substitution corresponded to bottle-grade and amorphous PET, respectively, assuming 90% and 70% substitution ratio.

#### 3.4.3 Beverage cartons

Beverage cartons consist of 75% paper, 21% plastic (as mixed foils) and 4% aluminum [27]. They are collected mixed with either mixed plastics or plastic bottles collected as curbside collection, from collection points or at some retailers. As no elemental composition of used beverage cartons exists, the thermal treatment was approximated with 75% paper, 21% mixed plastics and 4% aluminum.

##### 3.4.3.1 Collection and sorting of beverage cartons

The collection and sorting was approximated using the inventories for mixed plastic recycling, as they are collected mixed and undergo the same sorting routine.

##### 3.4.3.2 Recycling of beverage cartons

The recycling process has been newly modeled in this project. The sorted beverage cartons are dissolved into the individual components (fibers, aluminum and polyethylene). This dissolution process is carried out using a novel process which does not require any chemicals but needs sterilization of the beverage cartons beforehand with steam [7]. 97% of the fibers can then be fed to the cardboard recycling process (see chapter 1.1.1 cardboard recycling). The rejects (remaining fibers, aluminum and polyethylene) are collected, dried, and fed to the thermal utilization system. Data for the recycling process were derived from Model Group, Switzerland [7].

##### 3.4.3.3 Substitution

Beverage cartons are recycled to cardboard fibers which are later used in the corrugated board production. It was assumed, that they are used in the linerboard production, substituting primary fibers.

### 3.5 Life cycle inventories for metals

The following four metal fractions in municipal solid waste are reported and modeled individually: tinplate, other ferrous metals, used beverage cans (UBCs), and other aluminum packaging. Besides a material specific UBC collection system, the metals are collected mixed and later sorted using magnetic and eddy current separators [6]. In addition to the separately collected metals, also metal recovery and recycling from municipal solid waste incinerator bottom ash is modeled. While the ash treatment as such are described below, the recycling processes are assumed equal to the metal recycling from source separated factions where not stated differently.

#### 3.5.1 Life cycle inventories for tinplate recycling

About 13,000 t of tinplate cans per year are collected in Switzerland and further processed. Before being recycled, tin cans are detinned in Switzerland. The resulting tin can then be purified and be used to substitute primary tin. When tinplate is exported, it mostly enters the recycling without prior detinning. Most information in this chapter is taken from the report by Kägi and Zumstein [28].

##### 3.5.1.1 Collection of waste tinplate and aluminum

The first step to produce recycled and detinned material is to collect the scrap material. Data are based on information provided by the detinning company (Kägi and Zumstein [28]).

##### 3.5.1.2 Sorting of waste tinplate and aluminum

Before being detinned and recycled, the collected waste tinplate has to be sorted because the collected material consists of 77% waste tinplate and of 23% waste aluminum. The sorting process was modeled from primary industrial data on the diesel consumption and assuming an electricity consumption of 0.01 kWh/kg (based on ecoinvent dataset on the sorting of iron in Europe). The capital equipment was modeled based on the newly inventoried waste preparation facility in Switzerland.

##### 3.5.1.3 Detinning of waste tinplate

Data for detinning was collected from Elektrozinn AG, the only existing detinning plant in Switzerland. The detinning process is performed by means of electrolysis, where tin cake (tin content 85%) and black plate (tin content < 0.03%) can be recovered from tinplate. Additionally, the process delivers sludge as well as plastic and paper waste, which is burned in cement kilns or municipal incineration plants.

##### 3.5.1.4 Production of secondary tin

The tin cake is transported to a recycling plant and further processed together with tin and lead containing sludge from copper recycling. The lead contents are removed by vacuum distillation and high-grade tin (99.9%) is produced. Due to lack of data, the energy demand to produce 1 t of pure tin was estimated with literature values ([29], 20 MJ from 50% electricity and fuel oil, respectively). These values seem rather high, as they are valid for the processes “roasting, reduction & refining”, but data for the refining only are not reported.

#### 3.5.2 Life cycle inventories for recycling of low-alloyed steel

45% of the steel in Europe is produced using electric arc furnaces (EAF), mostly relying on scrap as the only iron bearing input [30]. The majority of ferrous scrap is, therefore, recycled in electric arc furnaces (EAF) which is the only steel production process applied within Switzerland. Therefore, only this process has been modelled. The dependency of electricity demand in EAF on the input material quality has been shown in Haupt et al. [31] and was implemented by assuming a 40% increase of energy consumption for ferrous scrap from basic bottom ash treatments. The advanced bottom ash treatment is assumed to also pretreat ferrous scrap which was found to substantially increase its quality [32]. While previously the EAF slag was landfilled, it increasingly replaces gravel in construction which was assumed in the new dataset. The emissions from utilized EAF slag have been assumed the equal to the emission in landfills and were included in the life cycle inventory accordingly.

##### 3.5.2.1 Collection of ferrous scrap

The collection of scrap differs largely depending on the scrap origin. The collection process has been assumed equal to the tinplate collection process described above.

##### 3.5.2.2 Recycling of ferrous scrap to low-alloyed steel

The direct smelting of iron-containing materials, such as scrap, is usually performed in electric arc furnaces (EAF). The major feed stock for the EAF is ferrous scrap, which may comprise of scrap from inside the steelworks, cut-offs from steel product manufacturers (e.g. vehicle builders), post-consumer scrap (e.g. end-of-life products) and scrap recovered from other waste treatment processes (e.g. the municipal solid waste incineration). The production of steel by the EAF process is a discontinuous process which involves raw material handling and storage, furnace charging with/without scrap preheating, EAF scrap melting, steel and slag tapping, ladle furnace treatments for quality adjustment, slag handling and continuous casting. The actual melting is done by lowering graphite electrodes into the scrap until they strike an arc that melts the scrap. More information on the individual steps is given in IPPC [33].

The steel produced in the process shown below is “low-alloyed”, and, therefore, contains a maximum share of 5% of alloying elements. This scrap is mostly used in the building industry. Ferrous scrap is generated in many processes and from a wide range of products. The scrap grades are defined in a national scrap nomenclature [34]. Tinplate from Swiss households that is recycled domestically, is detinned (Haupt et al. [6]). Tinplate leaving Switzerland for recycling is normally not detinned. Scrap from construction and demolition waste is often shredded or sheared before remelting but as these processes are largely depending on the origin of the scrap and are not used for scrap from households, they are not modelled below. The inventory below includes the remelting starting from scrap reception at the EAF in Switzerland and ends at billet casting.

After delivery, the ferrous scrap is temporarily stored in either an outdoor stockpile or directly in the hall where charges for heats are prepared. After ferrous scrap is brought into the preparation hall, the scrap is loaded into scrap baskets according to recipes. These recipes account for the different scrap qualities and are set up to yield a targeted output quality. The scrap is then melted using electricity and other energy carriers such as coal and natural gas. In addition, alloying elements are added and slag and dust are removed. Below, detailed information on the data sources, the emissions and waste are provided.

###### 3.5.2.2.1 Data sources

Swiss ferrous steel scrap is recycled in two electric arc furnaces (EAF) in Switzerland or in basic oxygen furnaces or EAFs abroad. The data is representative for recycling in Switzerland. Most information in this chapter was collected at an electric arc furnace in Switzerland.

No data has been found on the infrastructure needed for the process. Therefore, the ecoinvent process “market for electric arc furnace converter {GLO}” has been used, assuming the same amount as in previous European datasets.

###### 3.5.2.2.2 Emissions

Relevant air emissions of heavy metals vary largely depending on the scrap quality in electric steel making. Also varying with the scrap quality are chlorinated hydrocarbon emissions such as PCB and dioxins. Other VOC emissions can be remarkably high and correspond with the use of coal. Unfortunately, only annual data was available and was used in the processes below. The overall particle emissions were measured but the size distribution is unknown. According to Classen et al. [35], 42.5% of particles were assumed to be smaller than 2.5 μm, 42.5% of particles are assumed to be between 2.5 μm and 10 μm and 15% of particles are assumed to be larger than 10 μm.

###### 3.5.2.2.3 Wastes

The main waste generated in EAF steel making is slag. Slag is formed from lime to collect undesirable components in the steel. Their composition slightly depends on the alloy and on the sub-process they are generated in. As in Classen et al. [35] differences were assumed not to be significant and therefore neglected. While slag previously was mostly landfilled, it is recycled today. The final destination is, however, currently unknown. The slag is expected to substitute gravel in the current calculation. As the application of slag, for example in road construction, can result in emissions to ground water, the emissions modelled in the previous ecoinvent dataset have been added to the dataset (based on the ecoinvent dataset “treatment of slag, unalloyed electric arc furnace steel, residual material landfill”).

The treatment of off-gases is often performed in bag filters. The amount of dust filtered can be found in the inventories. For the further treatment, the previous ecoinvent processes are used.

#### 3.5.3 Life cycle inventories for recycling aluminum scrap

The collection and sorting processes for mixed aluminum scrap are modelled using the datasets for tinplate as these materials are mostly collected mixed. Used beverage cans (UBCs) are partly collected separately, but also undergo a sorting similar to the mixed aluminum to ensure that no other metals than aluminum are included. Therefore, also UBC collection was approximated with tinplate collection and sorting.

The recycling processes of aluminum (recycling of mixed aluminum scrap to cast aluminum and the utilization of UBC in the wrought aluminum production) were taken from ecoinvent, as no primary data was available. Therefore, no detailed description is given here but readers should refer to the ecoinvent database [1].

### 3.6 Life cycle inventories for waste preparation facility

In order to include the infrastructure of scrap sorting and preparation facilities in Switzerland, a new infrastructure inventory was created. It is based on the already existing ecoinvent inventory “scrap preparation facility {RER}” but without the administration building of 50 × 50 × 10 m^3^ which seems highly overestimated for a production plant with a capacity of 10,000 t per year. The lifespan of the building is estimated to be 50 years.

### 3.7 Life cycle inventories for biogenic waste treatment

While some municipalities collect kitchen and garden waste as mixed biogenic waste, others only collect one of those fractions or collect them separately. As no data was available for the distribution to the different collection strategies but only total amounts, garden and kitchen wastes are assessed as mixed fraction in all processes below.

#### 3.7.1 Collection

The collection of biogenic waste is organized locally and the modes and distances in the collection are, therefore, subject to large regional differences. Unfortunately, data is not available, neither on the different collection schemes nor on the types of vehicles used or the distance driven. Therefore, the transport has been approximated with 20 km collection in a municipal solid waste collection lorry.

#### 3.7.2 Composting and anaerobic digestion

The composting and the anaerobic digestion of biogenic waste from households, including kitchen and garden waste, were modelled based on Dinkel et al. [36] and Zschokke and Schleiss [37].

##### 3.7.2.1 Substitution

The utilization of biogenic waste in composting and anaerobic digestion yields nutrient rich secondary products (compost and digestate, respectively). Besides the substitution of fertilizer, the use of compost on land can have further benefits in terms of increased water retention of the soil (reduced irrigation), improved soil structure, and reduced erosion. These effects, that are largely dependent on local conditions, have been modelled for Switzerland by defining a humus-equivalency factor which is used to calculate the substitution of straw and peat by compost or digestate [37]. In addition, the substitution of N, P and K fertilizers were modelled taking the current mix of garden and kitchen waste into account [38], [39].

The utilization efficiencies of the organic and mineral nutrients depend on different factors such as the mineralization rate after application, and the type of soil and crop. The substitution ratios thus depend on the substitution efficiency which was assumed to be 20% for nitrogen, 90% of phosphorous and 100% for potassium [39] for both, digestate and compost.

### 3.8 Life cycle inventories for municipal solid waste incineration

Mixed municipal solid waste is collected from the curbside in most municipalities in Switzerland. The collection has been modeled based on the available processes in ecoinvent (details given below). For the modeling of the input dependent life cycle inventories of the waste treatment in municipal solid waste incineration, the LCA4waste tool was used which allows for assessing the input-specific inventories for all individual material fractions delivered to the municipal solid waste incineration plant [40]. The process of the thermal incineration and the later valorization of the residues was split into 3 process models and two energy flows: First, the incineration process, taking process- and input-specific emissions and the infrastructure into account, was modeled. Second, the material recovery from bottom ash was modelled for a basic and an advanced bottom ash treatment. While 80% of the bottom ash is assumed to be treated in basic bottom ash treatments, 20% are assumed to be treated in an advanced bottom ash treatment with higher material recovery rates. Third, for fly ash, modules for final deposits in underground deposit and residual material landfills as well as fly ash treatment in acid washing processes with and without direct zinc recovery were modelled (FLUWA and FLUREC, Schlumberger [41] and Boesch et al. [40]). The energy recovered (modelled as heat and electricity) are modelled directly in the product-process-matrix of the material flow analysis [2] based on the efficiencies provided in Table 1. A description of these flows is also given below.

**Table 1:** Efficiencies of municipal solid waste incinerator archetypes for reference year (2012) and future scenario (taken from Meylan et al. [42] for best case scenario for 2035).

Municipal solid waste incineration archetype	Heat recovery efficiency		Electricity recovery efficiency	
	2012	Scenario 5	2012	Scenario 5
Process steam	34%	60%	15%	29%
District heating	53%	61%	12%	28%
Combined heat and power, low eff.	25%	45%	17%	33%
Combined heat and power, high eff.	24%	70%	16%	21%
Electricity production	21%	30%	21%	27%

#### 3.8.1 Collection

As no data on the collection distance was available, a transport over 20 km in a municipal solid waste collection lorry was assumed, using the related ecoinvent transport process for Switzerland.

#### 3.8.2 Thermal treatment

The thermal treatment was modeled using the compositions reported in the Supplementary material and the LCA4waste tool [40], assuming a grate furnace, wet fly ash treatment, SNCR technology and a de-dust treatment based on an electrostatic filter.

#### 3.8.3 Energy recovery

The Swiss municipal solid waste incineration plants have been grouped into five archetypes to enable the modelling of different energetic efficiencies without regionalized modelling of the waste input and a spatially explicit modeling of the incineration plants. Please refer to Meylan et al. (submitted) for details of the municipal solid waste incineration archetypes formulated. Below, the most important information is provided. Table 1 then provides the efficiencies of the described archetypes in 2012 and for the future scenario in 2035 [42].

##### 3.8.3.1 Process steam (PS)

Five municipal solid waste incinerators are optimized to supply mainly steam to industrial processes all year around. Heat for district heating networks and electricity are regarded as “by-products” of waste incineration.

##### 3.8.3.2 District heating (DH)

Four municipal solid waste incinerators provide the base load of district heating networks for residential areas and office spaces. The highest efficiencies of these plants are reached in winter. Seasonal variation of heat recovery efficiency can be large if heat is mostly used for heating. Electricity production remains low in all seasons.

##### 3.8.3.3 Combined heat and power production, low efficiency (CHP)

Eight municipal solid waste incinerators are optimized for heat recovery and power generation (CHP plants). These municipal solid waste incinerators focus on heat in winter and electricity in summer resulting in seasonal variations for heat and electricity (opposed). The group of combined heat and power plants is divided into a group with low efficiencies (CHPlow) and high efficiencies (CHPhigh).

##### 3.8.3.4 Electricity production (EP)

Nine municipal solid waste incinerators are optimized for the production of electricity from the recovered energy. From the leftover energy, heat or steam is produced and delivered to the neighborhood.

The substitution of heat, electricity and energy carriers by the produced equivalents from the waste management system is subject to large uncertainties. Considering the attributional perspective, electricity recovered from waste-to-energy plants was assumed to substitute the Swiss electricity mix.

The substitution of heat is modeled taking the current district heating network into account under the assumption that it would not be deconstructed if it was not being fed by sufficient heat from municipal solid waste incinerators. It is, therefore, assumed that heat fed into the district heating network substitutes the mix of energy carriers used otherwise within the network (51% natural gas, 4% light fuel oil and 31% wood in industrial furnaces and 14% from nuclear power plants (burden free), [43]). As this is a very sensitive assumption, also a scenario with the current overall energy mix used for heating in Switzerland (also including heating systems not connected to the district heating network) was modeled, including 25% natural gas, 50% light fuel oil, 9% of heat from heat pumps, 6% from electricity (assuming 100% efficiency) and 10% of heat from wood [44].

#### 3.8.4 Fly ash treatment

The fly ash from Switzerland is either deposited in residual material landfills (after stabilization with cement), underground deposits or is processed in an acid washing processes for metal recovery. The acid washing process can either be used to produce a metal containing hydroxide sludge, which is later treated in a Waelz kiln to recover the metals (FLUWA) or can be directly linked to a hydrometallurgical zin recovery process (FLUWA). Both processes have already been included in the LCA4waste tool, but auxiliary material usage has been adapted based on industrial data.

#### 3.8.5 Bottom ash treatment

The treatment of bottom ash in Switzerland is very advanced compared to other countries and includes not only basic treatments, such as the recovery of metals with magnetic and eddy current separators, but also includes metal upgrading on split tables and numerous sorting processes yielding a higher quality of the recovered materials. For 2012, a basic and an advanced treatment were modelled, assuming that 80% of the bottom ash is treated in a basic process and 20% in advanced treatments. The efficiencies for the metal recovery are given in Table 2. While data for the basic treatments are taken form Allegrini et al. [45], the advanced treatment was modelled using data from Brupbacher [46].

**Table 2:** Metal recovery efficiencies assumed for the basic and advanced bottom ash treatment. The metallic share describes the share of metals available as metals and not bound within mineral components.

	Extraction efficiency		Metallic share (for LCA4waste tool)
	Basic treatment	Advanced treatment	
Ferrous scrap	81%	81%	100%
Aluminum	53%	85%	100%
Copper	46%	85%	100%
Zinc	0%	50%	100%
Cadmium	0%	0%	100%
Lead	0%	0%	100%
Gold	0%	50%	100%
Silver	0%	50%	100%
Wolfram	0%	0%	100%
Neodymium	0%	0%	100%
Nickel/Chromium-Steel	81%	81%	100%

Based on Haupt et al. [6] and Roth [32], an additional energy demand (+ 40%) for ferrous metals recovered from basic bottom ash treatments was assumed.

### 3.9 Life cycle inventories for waste treatments in cement kilns

Plastic wastes and beverage cartons can be utilized as alternative fuels in cement kilns. The utilization reduces the amount of fossil energy carriers and the need for dedicated waste treatments. When plastics and beverage cartons were used, a substitution of coal was assumed, as coal makes up for 97% of the fossil fuel input in Switzerland [47]. No substitution of alternative fuels and raw materials was assumed as also only primary material substitution was modeled for the fractions recycled. The substitution is modelled as a substitution of coal corresponding to the heating value while accounting for changed emissions. For this, the substitution is assessed as the replacement of 1 kg of clinker produced with coal by 1 kg of clinker produced with plastic waste (inventories are given in the Supplementary material). The process was modeled for PE waste and is later scaled to the heating values of other fractions to model also the utilization of PP, PS, PET and beverage cartons.
